# Comment on: A systematic overview of prospective cohort studies of cardiovascular disease in sub-Saharan Africa

**Published:** 2012-06

**Authors:** Ru-Yu Yuan

**Affiliations:** Department of Cardiology, Second Hospital of Tianjin Medical University, Tianjin, People’s Republic of China

## Dear Sir

It was with interest that I read the article titled ‘A systematic overview of prospective cohort studies of cardiovascular disease in sub-Saharan Africa’ by Andre Pascal Kengne, *et al.*, which was published recently in this journal. In this excellent article, the author introduced the association between cardiovascular diseases and related risk factors by performing a systematic review. However, we feel the article did not cover all aspects of the relationship between cardiovascular disease and related risk factors.

Firstly, habits may be very different among different ethnic groups, which is obvious in China, consisting of 56 ethnicities. This would have affected the outcome of the study,^1^ and it would have been better if there had been further subgroup analysis.^2^

Secondly, socio-economic status^3^,^4^ may be different in different regions in sub-Saharan Africa. Many studies may have come from different levels of hospitals, such as community and central hospitals, which also means that the available medical interventions may have been different.^5^,^6^

Thirdly, it is evident that that the study did not include all cardiovascular risk factors.^7^-^9^ There was no classification of the selected risk factors.^10^

All of these factors may increase the differences between the studies and affect the results to a certain extent. There is undoubtedly a need for well-designed, prospective, cohort studies from sub-Saharan Africa to clarify these issues.
